# Self-Sensing Electromechanical System Integrated with the Embedded Displacement Sensor

**DOI:** 10.3390/s24134102

**Published:** 2024-06-24

**Authors:** Shuxian Wang, Shiyou Liu, Zuqiang Su, Linlin Liu, Zhi Tang

**Affiliations:** 1 The Institute of Advanced Manufacturing Engineering, Chongqing University of Posts and Telecommunictions, Chongqing 400065, China; suzq@cqupt.edu.cn (Z.S.); liulinlin@cqupt.edu.cn (L.L.); tangzhi@cqupt.edu.cn (Z.T.); 2 Chongqing Qingshan Industry Co., Ltd., Chongqing 402761, China; shiyouliu@163.com; 3 School of Computer, Chongqing University, Chongqing 400044, China

**Keywords:** self-sensing, position detection, electromechanical system, integration, embedded

## Abstract

Conventionally, the electromechanical system requires the installation of auxiliary displacement sensors and only the amount on the drive part and motion end, which increases volume, cost, and measurement error in the system. This paper presents an integrated measurement method with a sensing head, which takes the equal division characteristics of mechanical structures as part of the sensor, thus, the so-called self-sensing system. Moreover, the displacement is measured by counting the time pulses. The sensing head is integrated with the entire electromechanical system, including the driving, transmitting, and moving parts. Thus, the integration of the sensing part is greatly improved. Taking the rotary table as a special example, and the sensing head embedded into each part of the system, displacement information is obtained by the common processing system and fused by the adaptive weighted average method. The results of the experiment show that the fusion precision of each component is higher than only the motor position information as the feedback. The proposed method is a practical self-sensing technology with significant volume reduction and intelligent control benefits in the industry, especially suitable for extremely small and narrow spaces.

## 1. Introduction

Intelligent machinery is facilitated by integrating new materials, design concepts, and control mechanisms that enable the machine to operate at a dynamic optimal state. To develop an intelligent machine, integration architecture is a component of primary importance [1]. The complex electro-mechanical systems, such as the aircraft engine component, stand for multi-axis machining tasks. Repairing these parts increases the high demand due to their unique part characteristics. A larger number of sensors is needed to monitor the work status of components. However, it is difficult to assemble a large number of sensors using the conventional manual assembly method. Hence, the advantages of integration stand out.

Researchers work on different perspectives for achieving integration, such as designing new structures or developing new materials. Nowadays, temperature sensors, pressure sensors, and vibration sensors with small volumes are on the market. They are embedded in the measured body to achieve its function without affecting the characteristics of the measured object [2,3,4]. In addition, some new materials with perception and conversion functions have been developed, for example, optical fiber sensing materials, stretchable materials, and piezoelectric ceramics [5,6]. Material-enabled sensors are more easily embedded into the measured object, so smart bearings, manipulators, and aircraft were born [7]. Additionally, some integrated position detection methods, which we focus on, have been put forward. J. Z. Shi and H. M. Zhang proposed a novel integrated position measurement unit for stepping motors, which just like a small stepping motor, have a separate stator, rotor, and stator winding [8]. This not only increases the length and weight of the motor but also adds to the complexity of the signal-processing circuit. In addition, a Hall sensor is commonly mounted on the stator of the brushless direct current motor to detect the rotation speed. However, it is difficult to assemble the sensor due to the narrow air gap between the stator and rotor. Furthermore, this detection method has a low resolution [9]. Recently, sensor-less technology has been one of the most studied methods for the position detection of motors and bearings. Among them, the magnetic flux observer, signal injection, back electro-motive force, and online parameter identification are the most common methods [10,11,12]. Even though they contribute to the compact and integrated structure, these methods rely on the precision of model parameters. Therefore, it is meaningful to embed displacement sensors into the electromechanical devices to monitor their position.

The grating and magnetic encoder as the conventional position sensor requires a larger number of grating lines or magnetic pole periods. It decides that the sensor has a tiny volume or that replacing it with a special material would be impossible. Thus, it is commonly mounted on the rotational shaft but is not suitable for the occasion of small installation spaces or other special occasions. For the wind power industry, the large turn table is hollow to facilitate cutter advancing and retreating, so the position detection of the table is difficult to realize by using slide-follower sensors. To solve the problem, strip grating is proposed to be stuck on the basic circle [13]. However, for applications with high circle precision requirements, introducing the edge error caused by the joint of the strip grating and not working well in the harsh work environment, strip grating in the application process also causes many problems. In addition, a commercial large bearing developed by the INA Bearing Company records the magnetic signal on the bearing surface [14]. The price is high, and the resolution is limited by the complex division circuit. Time grating is a new displacement sensor that builds the relationship between spatial displacement and time standards through a highly stable motion with orthogonally alternating physical fields such as the electric field, magnetic field, and optical field [15,16,17]. It transforms the space position into the time difference and obtains the position information by counting the time pulses. These kinds of time grating sensors have high precision and high resolution, and the flexible structure includes capacitive electrodes, coil winding, and a sine-shaped euphotic surface.

To be well-utilized in extreme environments such as space constraints, ultra-large components, hollow casings, and other harsh situations, this paper proposes a flexible sensing head and takes the self-structure of the measured object as a part of the sensor for integration. The embedded sensing head integrates with the measured object as a whole, just like an eye to the machine, which reduces the volume and increases the roost of the electromechanical system. Similarly, for a blind person, an eye combined with the brain is more coordinated and effective than a guide dog. That is because the brain, senses, and bodies of a person are highly integrated, even though the dog has keener vision, hearing, and smell. Thus, cooperation of all organs not only significantly reduces the burden of the person’s working process and simplifies the structure but also shortens the path of communication between the sensory organs, limbs, and brain. They must be integrated so that the person can respond to the measured external information directly, quickly, and accurately. Every part of the self-sensing electromechanical system with an embedded sensing head proposed in this article also achieves such integration.

## 2. Working Scheme of the Integrated Electromechanical System

### 2.1. Detection Principle of the Electromechanical System

**Figure 1:**
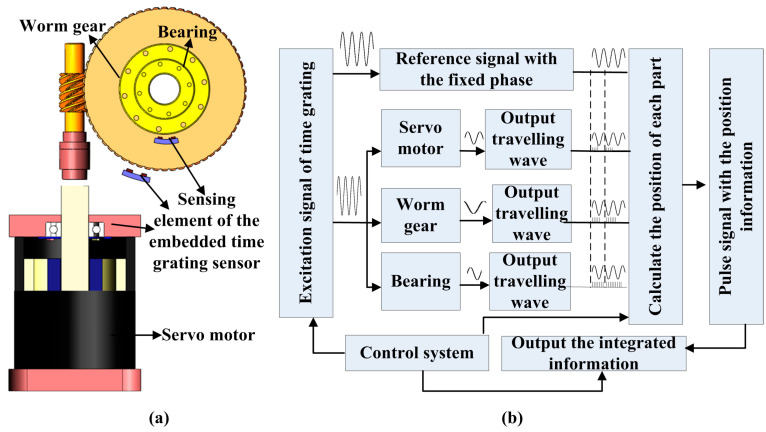
Working scheme of the electro-mechanical system with embedded position detection (take the NC rotary table as an example): (**a**) the structure of the integrated electromechanical parts; (**b**) the block diagram of the measuring principle based on the embedded time grating sensor.

According to the sensing coil or magnetic element, the embedded sensing sensors with flexible structures are embedded into the traditional electromechanical system to integrate with the measured part. Starting with a simple, generic numerical control (NC) rotary table, the proposed scheme for the integrated position detection is shown in Figure 1. The NC rotary table consists of three parts, including the mechanical drive, transmission, and motion, which are typified by the motor, worm gear, and bearing, respectively. Taking advantage of the equal space indexing of the motor, gears, and steel balls; it achieves metering equivalence based on the embedded time grating. Due to the position detection of each electromechanical component being realized by the embedded sensing element, hence, a common processing system can be used to provide the same excitation signal and clock frequency to measure the spatial position of different components, as shown in Figure 1b.

**Figure 2:**
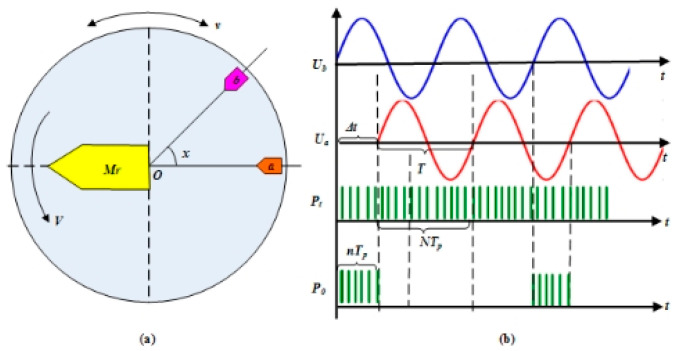
The basic working principle of a time grating sensor is the (**a**) measurement model (**b**) displacement obtained by counting the time pulses.

From the point of the voltage signal, the signal *U_a+_* is obtained by the embedded sensing head, which, travelling along the time axis, is taken as the moving reference system, and the static excitation signal *U_0_* is employed as the reference signal, as shown in Figure 2. They have the same amplitude and frequency value. As the measured object moves, there is a phase difference between the two signals that corresponds to a time difference Δ*t*, which can be determined by counting high-frequency time pulses with a period of Δ*P_t_*; the displacement can be expressed by

(1) $$X=W(\frac{m}{p}+\frac{\Delta t}{T})=W(\frac{m}{p}+\frac{nT_{p}}{NT_{p}})=W(\frac{m}{p}+\frac{n}{N})$$

where, *m* represents the number of cycles(0 < *m* ≤ *p*), *p* is the polar logarithm, *n* is the number of pulses with phase difference, *N* is the number of pulses corresponding to a unit cycle, and *T_p_* is the period of high-frequency pulses. Here, the time pulse sequence can be taken as a grating of time for the displacement measurement unit. As a result, the precision of measurement can be improved by the higher frequency of time pulses, which removes the height requirement for precision ruling and the structural limitation of the small pole pitch.

Based on this special electromechanical system with the embedded sensing head, a more intelligent and coordinated control is designed. This differs from the traditional closed-loop position detection method in which the position sensor is installed on the moving part and provides feedback on the position information to the servo motor. In this paper, an adaptive weighted averaging method is used to compensate for the position detection error by fusing the different measurement accuracy derived from the differential electromechanical part. Thus, an integrated electromechanical system is constructed using the same position detection and control method.

### 2.2. Multi-Head Information Fusion Technology Based on Adaptive Weighted Average

**Figure 3:**
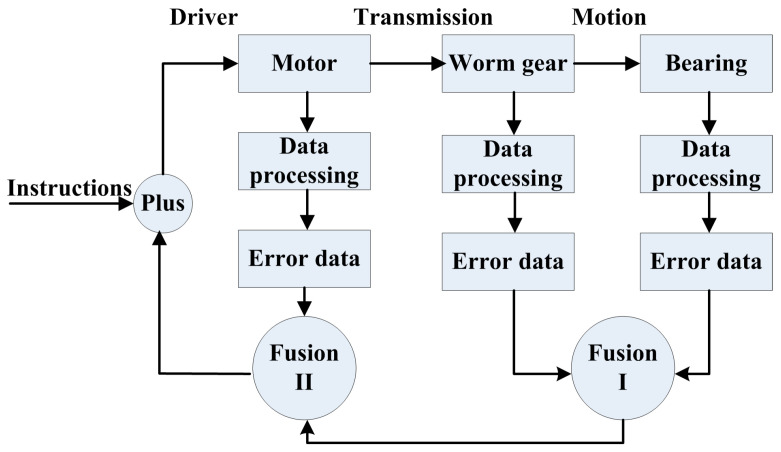
Fusion of the detection data based on the coefficients of adaptive weighted averaging.

The positioning of the turntable uses a dynamic sampling method. However, traditional data fusion is always commonly used for repeating the measurements of fixed points, which is not suitable for dynamic measurement. Thus, we make use of fusing the detection error data at different times and spatial locations to improve the positioning accuracy, as shown in Figure 3.

Each component has a differential detection accuracy. Compared with the bearing and worm gears, the servo motor rotates at a high speed and has low accuracy. Firstly, the dynamic data with a certain sampling interval are obtained by inputting positioning instructions. The error between the detection position and the theoretic position is then known. After fitting and correcting the error curve, there is a series of error data, mainly containing random errors. Assuming that the position detection errors of the worm wheel, bearing, and motor are set to *X*_1_, *X*_2_, and *X*_3_, respectively, The number of data points expressed is *m*. At the same sampling rate and time interval, the data points for worm gear and bearing are more than those of the high-speed motor. The values of the mean and standard deviation can be calculated by Equations (2) and (3).

(2) $${\bar{X}}_{j}=\frac{1}{m}\sum_{i=1}^{m}X_{ji},i=1,2,\ldots ,m;j=1,2,3$$

(3) $$\sigma_{j}=\sqrt{\frac{1}{m}\sum_{i=1}^{m}{\left(X_{1j}-{\bar{X}}_{j}\right)}^{2}}$$

Firstly, the worm gear and the bearing with relatively high precision are fused, and the fused error data are *Y*_1_. According to the transmission ratio, *Y*_1_ must be converted to data for high-speed conditions. Then the final data *Y*_2_ are obtained by fusing the *Y*_1_ and the detection error data *X*_3_.

(4) $$\left\{\begin{array}{c} Y_{1}=k_{1}X_{1}+(1-k_{1})X_{2}\\ Y_{2}=k_{2}Y_{1}+(1-k_{2})X_{3} \end{array}\right.$$

where *k*_1_ and *k*_2_ are the adaptive weighting factors for two fusion methods and satisfy the contains 0 < *k*_1_ < 1, 0 < *k*_2_ < 1. That can be expressed by the equations as follows.

(5) $$k_{1}=\frac{\sigma_{2}^{2}}{\sigma_{1}^{2}+\sigma_{2}^{2}};k_{2}=\frac{\sigma_{3}^{2}}{\sigma_{y}^{2}+\sigma_{3}^{2}}$$

where, *σ*_1_, *σ*_2_, *σ*_3_, and *σ_y_* are the standard deviations of the measurement data.

The positioning accuracy can be further improved by adding the inputted positioning instruction and the fusion error data *Y*_2_. Additionally, the system can also be monitored by judging whether the deviation value exceeds a certain range. If it does, we shut down the system immediately and carry out the maintenance to ensure the normal operation of the system.

## 3. Prototype of Embedded Sensing Head

Based on the sensing coil or magnetic element, a prototype of an electromechanical system is created by combining this part with the self-structure of the electromechanical components. Micro-processing of the component with different structures and materials is the key to designing the embedded time grating.

### 3.1. Embedded Sensing Head Based on Coils

**Figure 4:**
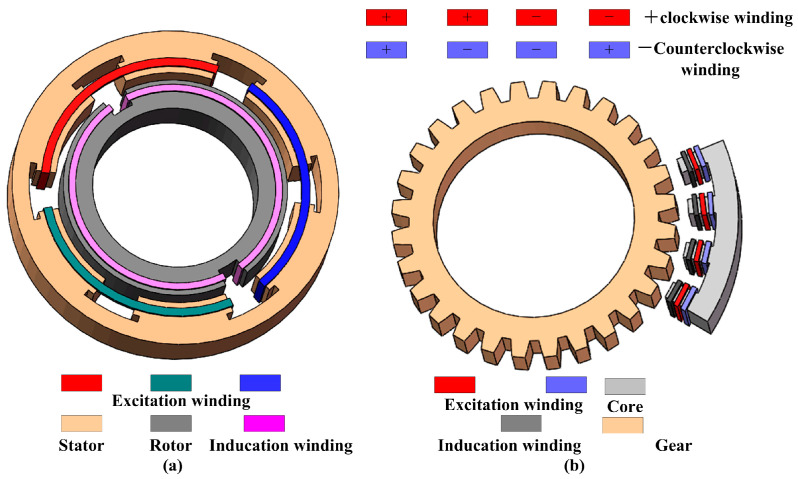
The embedded time grating sensor based on the sensing coil: (**a**) the induction coil is winding on the rotational part and the excitation coil is winding on the static part; (**b**) the excitation and induction coils are all winding on the static part.

Based on the law of electromagnetic induction, a coil is the most commonly used magnetic sensor. The sensing unit based on a coil is mainly composed of an excitation coil, an induction coil, an iron core, and a rotating body with a self-cogging structure (here called gear). Two kinds of different structures are shown in Figure 4. As shown in Figure 4a, according to the motor structure, three-phase excitation coils are wound around the static part of the electromechanical devices and the rotating body with an induction coil around the teeth, which is most applied to the actuator with the self-cogging structure. Based on the working principle of time grating, it needs a moving field with a far higher speed than the moving reference system. Thus, the excitation coils are excited by the high-frequency voltage, and the induction coil outputs a traveling signal. Figure 4b shows another differential structure. It takes the measured object with a self-cogging structure as the rotor of the sensor, and the sensing element consists of excitation coils, induction coils, and a common core. This structure avoids the sensing element separated by a quarter of pitch and replaces it by using control of the winding method to realize the spatial orthogonality, which makes a smaller volume and has obvious advantages when applied to space-starved applications. Of course, two secondary windings of SIN or COS signals are spatially displaced by 90°, which can also achieve the measurement.

Different from the common inductive sensor using the change of inductance to measure the displacement, the proposed embedded method processes the traveling voltage signal, which contains the displacement information. The relative displacement of the core and the gear result in a periodic change in air-gap magnetic permeance (AGMP). The AGMP varies between a minimum value when the core is aligned with the tooth and a maximum value when the teeth are unaligned. We consider the constant and fundamental component, the AGMP, and the displacement x, which can be mathematically expressed as

(6) $$\Lambda =\Lambda_{0}+\Lambda_{m}\cos(2\pi \frac{x}{W})$$

where Λ_0_ is the constant component, Λ*_m_* is the amplitude of the fundamental component, and *W* is the tooth pitch.

As shown in the winding and arrangement of coils in Figure 4b, With the excitation coil applied by altering the current signal, the output voltage of the induction coil is given by Equations (7)–(11).

(7) $$\begin{array}{l} E_{1}=-N_{2}\frac{d\Phi }{dt}=-N_{2}\frac{d(N_{1},I\Lambda )}{dt}=-N_{1}N_{2}\Lambda \frac{d(I_{m}\sin\omega t)}{dt}\\ \;=-k(\Lambda_{0}+\Lambda_{m}\cos(2\pi \frac{x}{W}))\cos\omega t \end{array}$$

(8) $$E_{2}=-N_{2}\frac{d\phi }{dt}=-k(\Lambda_{0}+\Lambda_{m}\cos(2\pi \frac{\left(x+(3/4)W\right)}{W}))\cos(\omega t)$$

(9) $$E_{3}=N_{2}\frac{d\phi }{dt}=k(\Lambda_{0}+\Lambda_{m}\cos(2\pi \frac{x+(6/4)W}{W}))\cos(\omega t)$$

(10) $$E_{4}=N_{2}\frac{d\phi }{dt}=k(\Lambda_{0}+\Lambda_{m}\cos(2\pi \frac{x+(9/4)W}{W}))\cos(\omega t)$$

(11) $$E_{s}=E_{1}+E_{2}+E_{3}+E_{4}=-2\sqrt{2}k\Lambda_{m}\cos(\omega t)\sin(2\pi \frac{x}{W}+\frac{\pi }{4})$$

where *N*_1_ and *N*_2_ are the turns of the excitation and induction coils, respectively. *Φ* is the magnetic flux, *ω* is the angular frequency of the current, *I_m_* is the amplitude of the excitation current, *k* is a constant, and *W* is the tooth pitch.

Here, we take the sensor with two group coils as an example. To construct a moving coordinate system, the other group excites a winding spaced distance *d* via the various winding methods, which contain *d* = (*m* + 1/4)*W*, *m* = 0, 1, 2, … Here, take *d* = (3/4)*W* as an example to illustrate. They have the same characteristics, except the two excitation signals have a 90-phase difference. Similarly, the induction voltage can be expressed by

(12) $$E_{1}^{\prime }=-N_{2}\frac{d\phi }{dt}=-N_{1}N_{2}\Lambda \frac{d(I_{m}\cos\omega t)}{dt}=k(\Lambda_{0}+\Lambda_{m}\cos(2\pi \frac{x}{W}))\sin(\omega t)$$

(13) $$E_{2}^{\prime }=-k(\Lambda_{0}+\Lambda_{m}\cos(2\pi \frac{\left(x+(3/4)W\right)}{W}))\sin(\omega t)$$

(14) $$E_{3}^{\prime }=-k(\Lambda_{0}+\Lambda_{m}\cos(2\pi \frac{\left(x+(6/4)W\right)}{W}))\sin(\omega t)$$

(15) $$E_{4}^{\prime }=-k(\Lambda_{0}+\Lambda_{m}\cos(2\pi \frac{\left(x+(9/4)W\right)}{W}))\sin(\omega t)$$

(16) $$E_{c}=E_{1}^{\prime }+E_{2}^{\prime }+E_{3}^{\prime }+E_{4}^{\prime }=2\sqrt{2}k\Lambda_{m}\sin(\omega t)\cos(2\pi \frac{x}{W}+\frac{\pi }{4})$$

The total output voltage *E* can be obtained by connecting the windings in series.

(17) $$E=E_{s}+E_{c}=2\sqrt{2}k\Lambda_{m}\sin(\omega t+2\pi \frac{x}{W}+\frac{\pi }{4})$$

The displacement is incorporated into the phase of the traveling signal, and it can be computed by counting the time pluses using the time grating.

### 3.2. Embedded Sensing Head Based on Magnetic Element

**Figure 5:**
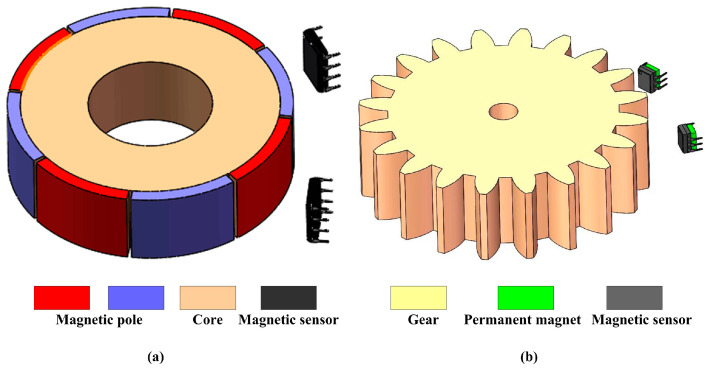
The embedded time grating sensor based on the magnetic sensor: (**a**) the actuator with the built-in magnetic ring; (**b**) the actuator with the self-cogging structure.

The rotational magnetic ring or the rotational gear matched with the static permanent magnet can also produce a uniformly moving magnetic field. Therefore, take the built-in magnetic ring, the self-cogging structure, or self-ball structure, and the electromagnetic actuators as part of the sensor. Additionally, using a magnetic sensor instead of the sensing coil could directly induce the changing magnetic field without the trouble of winding a coil, as shown in Figure 5. The sensitive direction of the magnetic sensor is along the direction of the magnetic ring rotation. When the center of the magnetic ring is facing the magnetic sensor, the minimum magnetic field strength along the sensitive direction is low. Similarly, the maximum magnetic field strength as the alternation of the two poles is face to the magnetic sensor, as shown in Figure 5a. Figure 5b shows the actuator with self-gear, which is made of the magnetism material. The spinning gear shapes a steady magnetic field created by a permanent magnet, such as the black bias magnet behind the magnetic sensor. When the sensitive direction of the magnetic sensor is parallel to the rotational direction, the magnetic field strength at the sensor position is low as a cog is in front of the sensor. Similarly, if a gap is in front of the sensor, the field strength is high. Thus, based on these two embedded sensing head structures, the changing magnetic field obtained by the motion of the electromechanical components can be simplified to a sinusoidal variable magnetic field.

Taking the linear magnetic element with a single sensitive direction as an example, two pairs of magnetic sensors are excited by sine or cosine altering voltage and separated by at least a quarter of the pole pitch or tooth pitch.

The output signals of the magnetic sensor can be expressed as follows.

(18) $$\begin{array}{ll} U_{1} & =SU_{e1}H_{1}=SU_{m}H_{m}\sin\omega_{e}t\sin\omega_{h}x\\ & =K\sin\omega_{e}t\sin\omega_{h}x \end{array}$$

where *S* is the sensitivity of the magnetic sensor, *U_e_*_1_ is the excitation signal, *H*_1_ is the magnetic field strength, *K* is a constant, and *H_m_* and *ω_h_* are the amplitude and angular frequency of the magnetic field strength, respectively. *U_m_* and *ω_e_* are the amplitude and angular frequency of the excitation signal, respectively.

Similarly, the output signal of the other magnetic sensor can be expressed by

(19) $$\begin{array}{ll} U_{2} & =SU_{e2}H_{2}=SU_{m}H_{m}\cos\omega_{e}t\cos\omega_{h}x\\ & =K\cos\omega_{e}t\cos\omega_{h}x \end{array}$$

where *U_e_*_2_ is the excitation signal, and *H*_2_ is the magnetic field strength.

The traveling wave can be obtained by adding two voltage signals together.

(20) $$U=U_{1}+U_{2}=K\sin(\omega_{e}t+\omega_{h}x)$$

Moreover, the displacement can also be measured by counting the time pulses.

## 4. Experiment Results and Analysis

### 4.1. Self-Sensing Electromechanical System

**Figure 6:**
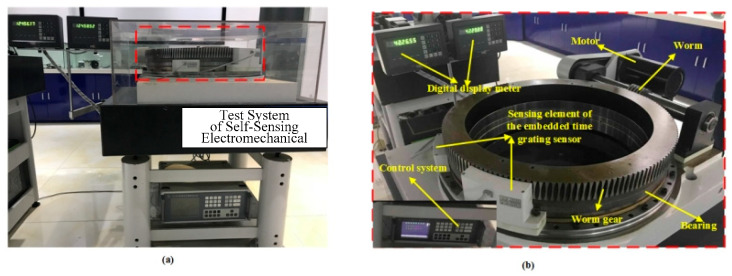
Prototype of the self-sensing electromechanical system (**a**) illustrated in a global view (**b**) partial detail.

The prototype of the electro-mechanical system is shown in Figure 6. The system consists of the typical components such as PMSM, worm gear (the transmission ratio is 160:1), and bearing, all with the embedded time grating sensor based on the sensing coil or magnetic sensor. The outer ring of the bearing is fixed on the marble platform, and the inner ring is connected to the worm gear. The motor drives the worm gear to form a rotary working system. The sensing element is packaged and fixed on the seat by the bracket. The control system collects the data from the worm gear and bearing to control the motor’s rotation. Also, the data for the worm gear and the bearing are shown on the digital display meter. For different electromechanical components with the embedded time grating sensor, no additional displacement sensor is needed. Based on this, an integrated electromechanical system with a common processing system is being built, which will make it possible to miniaturize complex systems and improve the performance of electromechanical systems.

### 4.2. Results and Discussion

**Figure 7:**
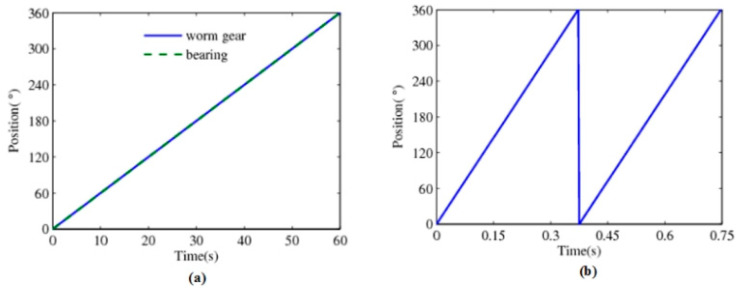
Position information: (**a**) the worm gear and the bearing; (**b**) the servo motor.

The experiments are carried out based on the prototype of the self-sensing electromechanical system. The important parameters in the test are as follows: The excitation frequency is 400 Hz, the sampling rate is 800 S/s, and the motor rotation speed is 160 rpm. Of course, the maximum excitation frequency can reach 40 kHz. Control the turntable so it runs at a speed of 1 rpm. That means collecting the measurement data points of the worm gear and bearing at a 0.015° interval and collecting data from the motor at a 2.4° interval. The position curves measured by the embedded time grating sensor for the bearing and worm gear in 60 s and the motor in 0.75 s are shown in Figure 7a,b. As shown in the figures, the detection data obtained from the worm gear and the bearing almost coincide due to their synchronous rotation. While the motor is rotating at a higher speed, it has rotated two circles in 0.75 s.

**Figure 8:**
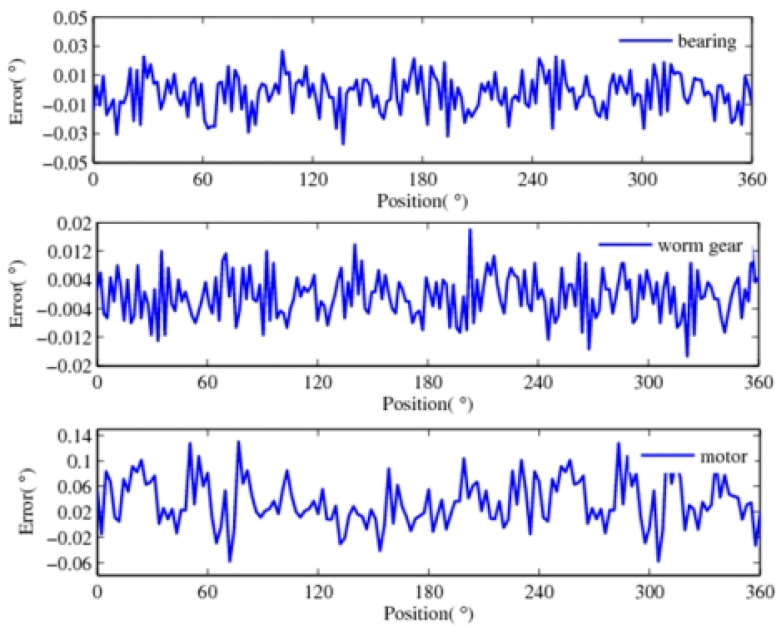
Detection error data obtained after correcting.

In the process of detection, the data errors between the measured data and the theoretical position are obtained. It mostly contains the system errors caused by mechanical processing, installation, and electrical errors. Thus, the error is corrected based on the amplitude–frequency characteristics of the error, and there are a large number of random errors remaining, as shown in Figure 8. The measurement accuracy of the bearing, worm gear, and motor can reach up to 0.06°, 0.024°, and 0.20°, respectively. The motor can reach up to 0.06°, 0.024°, and 0.20°, respectively. It can be seen that there is a great difference in the detection accuracy of the driver, transmission, and motion components.

**Figure 9:**
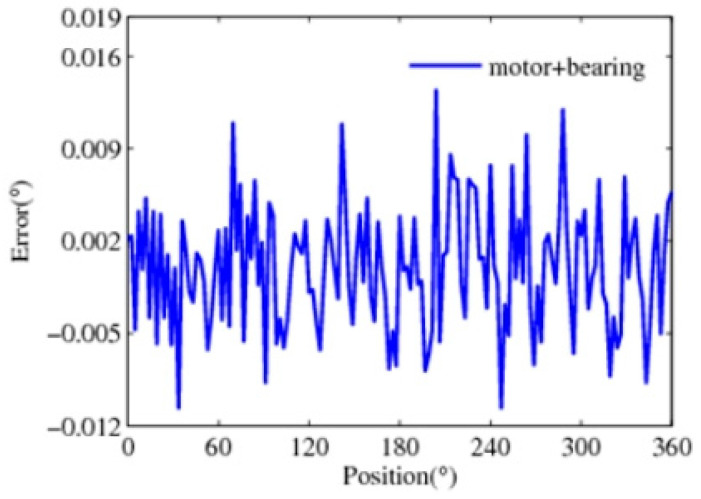
Fusion of motor and bearing detection error data.

Like the common closed-loop control used in the electromechanical system, we take the fusion error data of the motor and the bearing feedback to the instruction. According to the aforementioned adaptive weighted averaging algorithm, the relevant parameters are shown in Table 1. Figure 9 shows the obtained error curve after fusion. The error data after fusion can reach ±0.012°. Compared with the semi-closed-loop control, which only feeds the measurement error of the servo motor back to the instruction, the control precision is improved by approximately 8 times.

**Figure 10:**
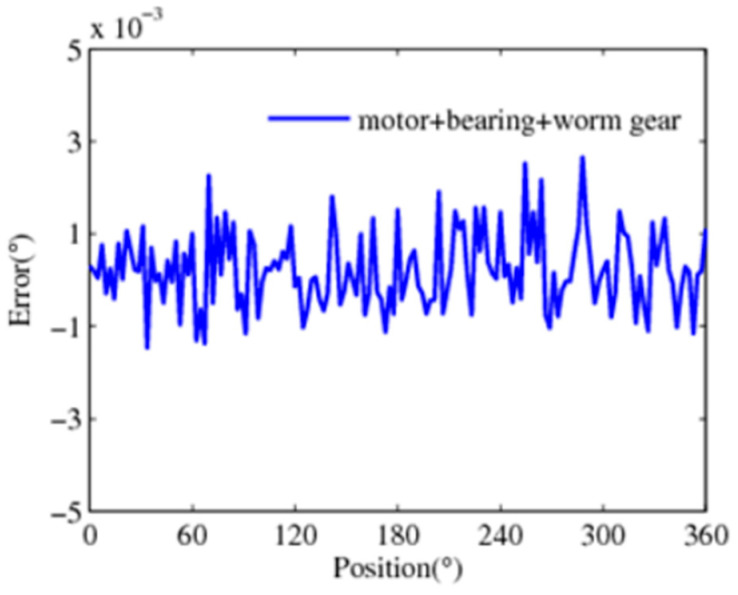
Fusion of errors in the motor, bearing, and worm gear.

The position information for each component of the electromechanical system is detected by a common processing system and improved by the adaptive weighted averaging method. Bearing and worm gear display synchronous motion with relatively higher precision, and the detection data for them are fused first. According to the transmission ratio, these results are converted to the high-speed end and fused with the detection error data of the motor. The relevant parameters are shown in Table 2, and the final error data are curved in Figure 10. In the range of measurable values, which is 360°,the error value of the three-error data fusion is 0.004°, which is nearly 48 times higher than that of the single motor detection as the feedback and nearly 6 times higher than the fusion of the motor and the bearing detection. This comparison shows that the error is greatly reduced by fusing the measurement error of each component. Adding the error after fusion and the instruction as the real positioning value, the precision of the positioning detection is greatly improved.

## 5. Conclusions

This paper proposes a self-sensing electromechanical system integrated with an embedded sensing head in each part. In the special NC rotary table, the motor, worm gear, and bearing are integrated as parts of the embedded sensing head with their mechanical equal division, such as gears, magnetic poles, and steel balls. Compared with the traditional feedback of position information only in the motor part, this multi-part integration method is convenient for fault diagnosis and improves control performance. That operation is closer to realizing the unification of the human brain to complete sensory integration, information fusion, and more coordinated action. Thus, the structure of the sensing head is flexible and can be integrated with mechanical systems by the sensing element discretization and miniaturization. Furthermore, the measurement principle of the proposed method is simple, and no extra sensors are needed. That requires the electro-mechanical system to work both as a sensor and possibly as an actuator. Additionally, the whole system will be more coordinated with the common processing system based on the adaptive weighted averaging method. The proposed method is low-cost and facilitates the online monitoring of position information for each component. Although we take the simple NC rotary table as an example, the embedded sensing head can be applied to other complex electro-mechanical systems as well. Of course, the calibration of sensors in embedded position measurement is a challenge, and related research such as self-calibration can be explored in the subsequent research work.

## Figures and Tables

**Table 1:** The relative parameter of the fusion data of the bearing and the motor.

	Average Value	Standard Deviation	Fusion Coefficient
bearing	−5.75 × 10^−4^	0.0386	0.9843
motor	0.0354	0.0049	

**Table 2:** Relative parameters of the error fusion of the bearing, worm gear, and the motor.

	Bearing	Worm Gear	Motor
Average value	−5.75 × 10^−4^	−0.0027	—
Standard deviation	0.0049	−0.0103	
Fusion coefficient	0.182		
Average value of the fusion data	−9.522 × 10^−4^		0.0354
Standard deviation of the fusion data	0.0041		−0.0386
Fusion coefficient	0.989		

## Data Availability

The data presented in this study are available on request from the corresponding author.
